# Mindfulness Is Associated With Lower Stress and Higher Work Engagement in a Large Sample of MOOC Participants

**DOI:** 10.3389/fpsyg.2021.724126

**Published:** 2021-09-10

**Authors:** Larissa Bartlett, Marie-Jeanne Buscot, Aidan Bindoff, Richard Chambers, Craig Hassed

**Affiliations:** ^1^Wicking Dementia Research and Education Centre, University of Tasmania, Hobart, TAS, Australia; ^2^Menzies Institute for Medical Research, University of Tasmania, Hobart, TAS, Australia; ^3^Centre for Consciousness and Contemplative Studies, Monash University, Melbourne, VIC, Australia; ^4^Faculty of Medicine, Nursing and Health Sciences, Monash University, Melbourne, VIC, Australia

**Keywords:** mindfulness, meditation, stress, work engagement, online course

## Abstract

**Objective:** This study aimed to understand the associations between mindfulness, perceived stress, and work engagement in a very large sample of English-speaking adults, from 130 different countries. It also aimed to assess participants' self-reported changes following a 6-week mindfulness massive open online course (MOOC).

**Methods:** Participants in the 6-week MOOC were invited to complete pre-post online surveys. Cross-sectional associations were assessed using univariate linear models, followed by structural equation models to test mediation pathways in baseline data (*N* = 16,697). Self-reported changes in mindfulness, stress and engagement following training were assessed using paired *t*-tests (*n* = 2,105).

**Results:** Each standard deviation unit increase in mindfulness was associated with a 0.52 standard deviation unit decrease in perceived stress, and with 0.06 standard deviation unit increment in work engagement. 73% of the influence of mindfulness on engagement was direct. Following the mindfulness MOOC, participants reported higher mindfulness (*d* = 1.16), reduced perceived stress (*d* = 1.00) and a small improvement in work engagement (*d* = 0.29).

**Conclusions:** Mindfulness was associated with lower perceived stress and higher work engagement in both cross-sectional and longitudinal analyses. These findings support mindfulness as a potentially protective and modifiable personal resource. The MOOC format offers a low cost, highly accessible means for extending the reach and potential benefits of mindfulness training to large numbers of people.

## Introduction

Mindfulness, or intentionally paying attention to current experiences with an open and non-judging attitude, is described as both a skill and as a way of being (Bishop et al., 2004). As a skill, mindfulness can be cultivated through training in both attention and attitude. Training attention takes place formally through regular meditation practice, and informally by intentionally giving attention to present moment internal and external experiences in day-to-day life. Regular and sustained mindfulness practice has been shown to improve attentional control, increase awareness of internal and external experiences and reduce automatic reactivity in emotional, physiological and behavioral domains (Chambers et al., 2009; Creswell and Lindsay, 2014; Garland et al., 2017a). Mindfulness practices also develop attitudinal qualities such as acceptance, openness, curiosity, compassion and non-judging (Crane et al., 2016). The skills and attitudes acquired through practicing mindfulness thus support a way of being that is characterized by intentional attentiveness, awareness and acceptance (Bishop et al., 2004).

Epidemiological research into the relationship between mindfulness and health and performance outcomes is emerging. Mindfulness correlates strongly with lower perceived stress, and moderately with positive subjective wellbeing in a sample of health professionals (*n* = 450; Atanes et al., 2015) and amongst university students (*n* = 135; Palmer and Rodger, 2009; *n* = 85; Zimmaro et al., 2016). In a larger sample of community-dwelling Swedish adults mindfulness is associated with lower stress, depression and anxiety, and positive health perceptions (*n* = 1000; Bränström et al., 2011). Further, cardiovascular health problems associated with elevated stress appear to be ameliorated by higher mindfulness (Loucks et al., 2015; *n* = 382). These correlational findings collectively support the premise that increasing people's mindfulness may lead to beneficial health and performance outcomes. However, the sample sizes and context-specificity for these studies somewhat limits generalisability. To establish mindfulness as a determinant, or predictor, of lower stress and of positive health and performance at population level requires more evidence from large and more broadly representative population samples. Whether correlates are directly or indirectly attributable to mindfulness should also be investigated.

Intervention research provides support for the hypothesis that increasing mindfulness can lead to health and performance benefits. Controlled studies show participants in mindfulness-based interventions (MBIs) consistently report lower perceived stress following training (Balconi et al., 2019; Colgan et al., 2019). Mental health, executive functioning and social behaviors are known to be detrimentally affected by high stress (Cohen et al., 2019), are also shown to consistently improve following mindfulness training (Gallant, 2016; Donald et al., 2019). Further, beneficial training effects for resilience (Joyce et al., 2018), cognitive functioning (Chiesa et al., 2011) and work engagement (Dane and Brummel, 2014; Vonderlin et al., 2020), indicate mindfulness may be a protective personal resource that can ameliorate the detrimental effects of stress and enhance health and performance.

Based on this collection of promising evidence, mindfulness training is being taken up in many health-related, educational and corporate settings (Reb and Choi, 2014). MBIs for working populations are proposed as a means to foster employee performance, relationships and wellbeing (Good et al., 2016). Work engagement, which comprises vigor, dedication and absorption is of particular interest to employers because it links personal wellbeing factors with work performance (Schaufeli et al., 2006; Burton et al., 2017). For example, in corporate environments higher work engagement correlates with outcomes such as task performance, and innovative work behavior (Gemeda and Lee, 2020) and in healthcare it contributes significantly to lowering costs while improving efficiency, quality of care, patient safety, physician satisfaction and retention (Perreira et al., 2018). Correlations between higher mindfulness and greater work engagement have been reported (Liu et al., 2019), however, most workplace-based MBI studies have focused on employee stress, mental health and wellbeing, few report organizational benefits such as performance and engagement (Bartlett et al., 2019).

Traditionally MBIs have been taught in individual or class-based face-to-face formats, and involve didactic and reflective interactions between the course participants and teacher (Crane et al., 2016). This direct and personal interaction allows for questions and real-time discussion to reinforce learning and address any difficulties arising as the participants learn to practice and apply mindfulness skills, including meditation. However, the face-to-face nature of individual or class-based learning presents an accessibility challenge for many people (e.g., West, 2011; Bartlett et al., 2016). Given restrictions on social gatherings and reports of escalating mental health problems due to the COVID-19 pandemic (Newby et al., 2020), an evidence-based approach to extending the reach and accessibility of potentially protective interventions such as mindfulness training via online delivery is both warranted and pressing.

Translating face-to-face mindfulness courses to an online medium presents an opportunity to reach large numbers of people, while limiting the associated increase in resource demands. Online learning is especially enabling for people who, through isolation or reduced mobility, cannot otherwise access face-to-face courses. It also presents a number of challenges however, including the lack of direct face-to-face interaction between participant and teacher, and the relative isolation of the online learning environment. These factors can limit the feasibility of responding appropriately to learners who are struggling with personal and health-related issues (Muilenburg and Berge, 2005).

Despite these challenges, there is an emerging literature demonstrating that MBIs delivered online can produce similar benefits to those delivered in face-to-face format (Platt et al., 2014). In the mindfulness field two meta-analyses have been published showing positive results for clinical and non-clinical populations following web-based MBIs (Spijkerman et al., 2016; Toivonen et al., 2017). Both reviews included MBIs with various formats and sample sizes (n = 13 to n = 257). Neither review found differences between synchronous (delivered in real time using media such as instant messaging platforms, telephone, or videoconferencing) vs. asynchronous (delayed delivery methods such as email or message boards), or facilitated vs. self-directed online formats. However, Spijkerman et al. (2016) noted stronger effects were common from online MBIs when therapist guidance was available for participants.

Recent years have seen growing use of massive open online courses (MOOC) for education and training purposes (Ebben and Murphy, 2014). MOOCs provide an interactive educational infrastructure that supports delivery of online courses to large numbers (e.g., thousands) of participants, commonly with a good deal of heterogeneity. The MOOC platforms generally combine didactic teaching, both in real-time and asynchronously, with peer-to-peer interaction (Sunar et al., 2017). Emerging evidence supports the effectiveness of the MOOC format for delivering health-related behavioral and educational interventions (Eccleston et al., 2019). However, to date there is little or no published evidence of the effectiveness of MBIs delivered using the MOOC format.

Using a very large baseline sample of MWPP-MOOC participants, the first objective of this study was to assess the associations between mindfulness, work engagement and perceived stress and to investigate the extent to which mindfulness influences engagement directly or indirectly (via lowering stress) (Aim 1). The second objective, drawing on pre-post intervention data, was to test the direction and magnitude of changes in mindfulness, stress and work engagement following participation in a MOOC based MBI (Aim 2).

## Methods

### Participants

The *Mindfulness for Wellbeing and Peak Performance MOOC* (MWPP-MOOC) is housed on the FutureLearn learning platform based in the United Kingdom. The course was in English but open to anyone around the world with the required English language skills and does not cost money to join. Participants self-select to do the course based on their interest in learning about mindfulness and recruitment into the course was via the FutureLearn website or newsletter, organic web search and word of mouth referrals. The platform allows for collection of basic demographic data such as age, gender and country of origin, but not for more personal health information such as mental health history. Participation in the research was optional and additional to course enrolment. Prior to course commencement, MOOC enrollees were invited to read the participant information sheet and provide their consent to having their anonymous data available to the course facilitators for research purposes.

### Procedure

The MWPP-MOOC was developed at Monash University in 2015 (Hassed and Chambers, 2015). FutureLearn is a British digital education platform founded in December 2012 and is jointly owned by The Open University and SEEK Ltd. As of March 2020, FutureLearn included over 250 UK and international partners in university, industry and government sectors. It therefore has a very broad reach for recruiting diverse learners from many countries, ages and educational backgrounds. Between 2015 and 2020, the MWPP-MOOC has run 14 times and enrolled nearly 400,000 participants. When enrolling in the course, participants can opt to complete pre- and post-training surveys, providing data from a large sample of non-clinical adult learners for research purposes.

The MWPP-MOOC is an asynchronous online mindfulness course developed and delivered by medical (CH) and psychological (RC) professionals, each with decades of experience in developing, contextualizing and delivering mindfulness training in educational, workplace and community settings. Both teachers have ongoing positions at a large Australian university where they deliver mindfulness training programs to over 6,500 people per year. The MWPP-MOOC includes up to 3 h of coursework per week, over 6-weeks. The course shares some similarity with the Mindfulness-Based Stress Reduction protocol, but has shorter meditations (5–10 min duration instead of 20–45 min). Weekly topics build progressively on one another and include (1) the formal and informal practice of mindfulness, (2) the role of mindfulness in stress reduction, (3) how it impacts upon work and study performance, (4) the role of mindfulness in self-compassion, communication and relationships, and (5) how to maintain and extend the practice after the end of the course. The MWPP-MOOC includes a variety of media such as short videos, carefully curated open-source articles, links to useful websites and other resources, downloadable guided meditations and quizzes to test knowledge and understanding of key principles. The course structure is detailed in the Supplementary Material.

MPWW-MOOC participants have access to online forums (moderated by CH and RC). The forums are semi-structured, relate to the content presented in that week, and are intended to encourage self-reflective learning, provide answers to questions, create a sense of community, and support learners. The forum moderators also guide communication quality and help learners to seek professional help if necessary. Unsolicited participant reports indicate the forums are a favorite aspect of the MOOC, providing a “live” feel to the course, informal social support and an opportunity to deepen learning.

Weekly feedback videos are another key feature and popular aspect of the MWPP-MOOC. These videos involve the facilitators engaging in informal discussion about key insights, topics and questions arising from that week's forums and further enhance the sense of responsiveness and interactivity between course facilitators and learners. Live engagement with the discussion boards, moderation, feedback videos and surveys closes 6-weeks after the course opens, although learners can retain access to the course materials for a further 2 weeks. Participants in the September 2015 and February 2016 MWPP-MOOCs provided data for the present study.

### Measures

Trait mindfulness was measured using the Freiburg Mindfulness Inventory (FMI; Walach et al., 2006). The FMI is a brief, 14-item, unidimensional measure of trait mindfulness that includes questions about the respondents' attention, attitude and awareness. Response options range from 1 (Rarely) to 4 (Almost always). One item is reverse-scored and responses are then summed for a total score (range 14 to 56). Internal consistency of the baseline FMI data in our sample was good (α = 0.90).

Perceived stress was measured using the Perceived Stress Scale (PSS; Cohen et al., 1983). The 10-item PSS was designed for use in community samples and subjectively measures participant appraisals of the degree to which life is stressful (unpredictable, uncontrollable and overloaded) (Cohen et al., 2007). Response options range from 0 (Never) to 4 (Very often) on a 5-point Likert-type scale. This measure is commonly used in MBI research, with strong negative associations between PSS and mindfulness scores (e.g., Manotas et al., 2014; Atanes et al., 2015; Bartlett et al., 2019). The PSS score is calculated by reversing four negatively worded items and then summing responses. Higher scores indicate higher perceived stress (range 0–40). Internal consistency for the PSS-10 in our sample was good (α = 0.89).

The Utrecht Work Engagement Scale (UWES; Schaufeli et al., 2002) was used to measure three dimensions of work engagement: vigor, dedication and absorption. Vigor is characterized by mental resilience and high energy levels while working or studying, and one's willingness to put effort into one's work or study. Dedication is characterized by a sense of involvement, significance, inspiration, challenge, enthusiasm, and pride. Absorption is characterized by high engagement with, and absorption in, work. The UWES has also been used previously to measure study engagement (Schaufeli et al., 2002), with an adapted version (UWES-S) subsequently published by the authors. We instructed anyone who identified as a student to consider their experience studying, rather than working, when completing the UWES. The UWES comprises 17 items measured on a 6-point Likert-type scale ranging from 1 (Almost never/A few times a year or less) to 6 (Always/Every day). Mean responses are computed for total and subscale scores (range 1 to 6). Internal consistency for the UWES in our sample was good at whole scale (α = 0.95) and factor level (vigor α = 0.86, dedication α = 0.92, absorption α = 0.86).

### Data Analysis

Data were collected at baseline and post-intervention using online surveys (SurveyMonkey) presented via links included in weeks one and six of the MWPP-MOOC. Prior to analyses, data from each of the three measures were standardized to z-scores, to support interpretability of results. Internal consistency for the outcome measures was assessed using Cronbach's alpha coefficient. Correlations between mindfulness (FMI), perceived stress (PSS) and engagement (UWES) data were inspected using Pearson coefficient.

To address Aim 1, Z-scores for each outcome were used to support comparison of the different measures. Univariate linear regression modeling was applied to test the direction and magnitude of cross-sectional associations between self-reported mindfulness, work engagement and stress in the large baseline data set. Graphical model checks were conducted to detect potential violations to model assumptions, including homogeneity of the variance, normality of the residuals and outlier datapoints. Structural equation modeling (SEM) using the product of coefficients method in the ‘lavaan‘ package (Rosseel, 2012) was then applied to estimate the extent to which the relationship between mindfulness and work engagement is mediated by perceived stress.

To address Aim 2 the difference in means from pre- to post-intervention was assessed using paired t-tests. Observations were included only when cases could be linked by IP address at both time points. First completed attempts only were included. To estimate the association between changes in mindfulness following course exposure with perceived stress and work engagement we regressed PSS and UWES scores post-training on FMI_*post*_ – FMI_*pre*_, adjusted for PSS and UWES pre-training. Cohen's *d* standardized mean difference effect estimates were computed and interpreted in line with recommendations, where an effect of 0.2 is weak, 0.5 moderate and 0.8 or above is strong (Lakens, 2013). We used Harman's single factor test to assess the degree to which common method variance (covariance between scales which can be attributed to the method of data collection) could bias our results (Fuller et al., 2016).

Analyses were conducted using the “Hmisc,” “psych,” and “lavaan” packages (Rosseel, 2012; Revelle, 2019; Harrell et al., 2020) in the R statistical computing environment (R Core Team, 2019), with significance at α = 0.05.

## Results

### Sample and Participant Characteristics

In the first two runs of the MWPP-MOOC there were 86,260 registered learners, with 20,331 consenting to research and 16,697 with complete data at baseline. The post-intervention surveys were completed by 4,681 participants, but not all of these cases could be confidently linked between time points. Complete pre-post data were available for 2,105 participants. The age of learners varied from 18 to over 65 years of age. The largest age bracket with 27% of learners was 56–65 years of age. Learners joined from 130 countries with most located in the UK (42%), Australia (22%), the USA (5%), Canada (2%), and Ireland (2%). In terms of gender, 76.4% of participants identified as female.

Mindfulness experience among learners varied with 58% having had no previous experience (academically or professionally) in mindfulness, 26% having taken another course in mindfulness and 11% working in a related field. Participant motivations for registering included dealing with stress or health problems or to improve work/study performance (e.g., efficiency, coping with pressure, reducing errors) and work prospects. Other reasons included pursuing an academic interest in mindfulness, looking for more enjoyment in life, developing retirement interests, wanting to slow down or to enrich relationships.

### Aim 1: How Are Mindfulness, Stress, and Work Engagement Related?

Correlations between mindfulness, perceived stress and work engagement were inspected in the large baseline sample (*n* = 16,697). The UWES subscales were strongly intercorrelated (all *r* = 0.81). The FMI data had a moderate sized positive association with UWES data at whole scale (*r* = 0.40) and subscale level (vigor *r* = 0.47; dedication *r* = 0.36; absorption *r* = 0.29), and a moderate to strong negative association with PSS data (*r* = −0.59). All these correlations were significant (*p* < 0.001). A single, unrotated factor explained 32% of covariance between items across all scales.

Univariate models (Table 1) showed strong evidence that higher mindfulness was associated with lower perceived stress. Each standard deviation unit increase in mindfulness was associated with a 0.52 standard deviation unit decrease in stress (*p* < 0.001), accounting for 34% total variability in PSS data. A single standard deviation unit increase for mindfulness was associated with 0.06 standard deviation unit increment in overall work engagement scores (p <0.001), accounting for 16% variability in UWES. Within the UWES subscales, vigor appears to account for more variation in mindfulness (22%) than dedication (13%) and absorption (8%). Each unit increase in perceived stress predicted a 0.06 unit decrement in overall engagement scores (*p* < 0.001), accounting for 13% variability in UWES.

**Table 1:** Univariate regression model results of the relationship between mindfulness, stress, and work engagement (*n* = 16,697).

**Independent variable**	**Dependent variable**	**β**	**95% CI**	***p***	***R* ^**2**^**
Mindfulness	Perceived stress	−0.52	−0.53, −0.51	<0.001	0.34
	Work engagement	0.06	0.06, 0.06	<0.001	0.16
	Vigor	0.07	0.07, 0.08	<0.001	0.22
	Dedication	0.07	0.06, 0.07	<0.001	0.13
	Absorption	0.05	0.04, 0.05	<0.001	0.08
Perceived stress	Work engagement	−0.06	−0.06, −0.06	<0.001	0.13

*Stress: PSS-10; Work Engagement: UWES; Mindfulness assessed using FMI; univariate linear regression model on standardized scale data with α = 0.05; β (effect size) per SD unit change in response for each SD unit increase in predictor; R^2^: percentage of response variability explained by the independent variable*.

**Figure 1:**
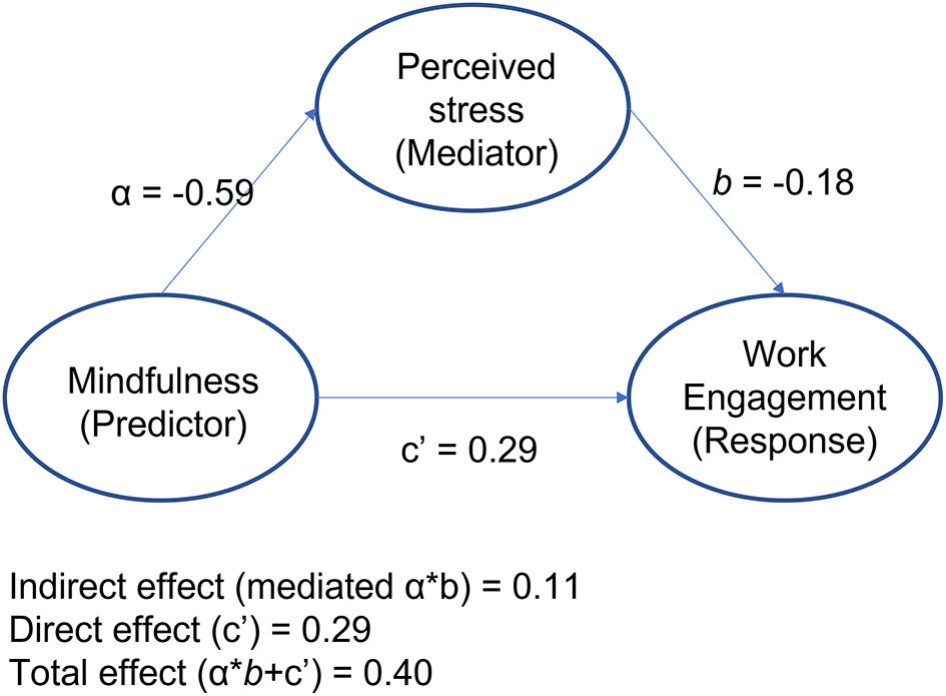
Mediation path showing direct and indirect effects of mindfulness on work engagement.

The mediation results are presented in Figure 1. In this cross-sectional path analysis, a unit increase in mindfulness was associated with higher work engagement (β = 0.29, SE = 0.009, *Z* = 33.16, *p* < 0.001) and lower perceived stress (β = −0.59, SE = 0.006, *Z* = −91.19, *p* < 0.001). While the effect of mindfulness on work engagement was mostly direct (73%), the total effect was partially (27%) mediated via lower perceived stress (β = −0.18, SE = 0.010, *Z* = −19.00, *p* < 0.001).

### Aim 2: Efficacy of the MWPP-MOOC

**Figure 2:**
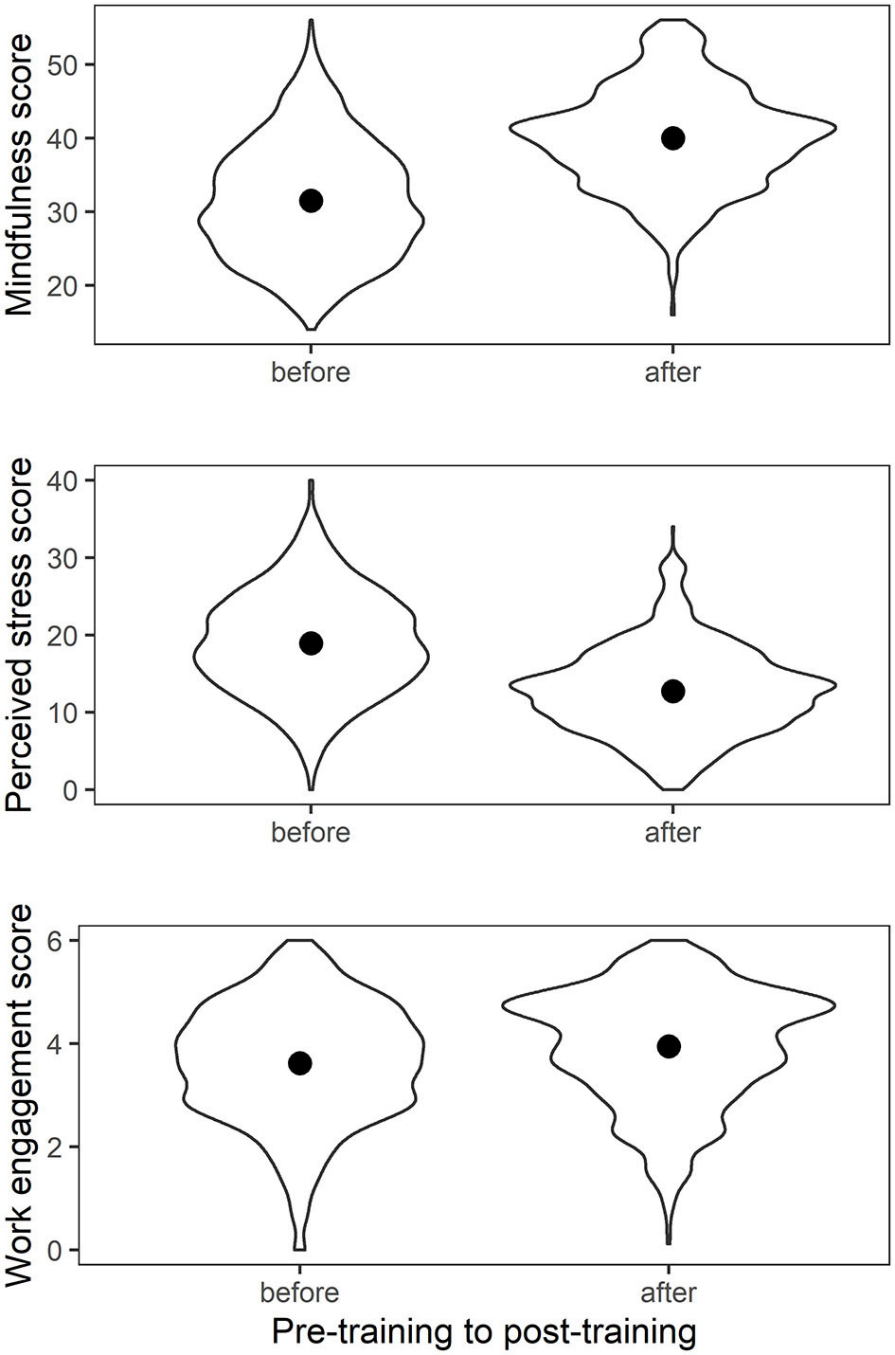
Difference in means from pre- to post-training for mindfulness, perceived stress, and work engagement. Violin plots show the kernel density of observations at each score on the Y axis reflected along the midline, to illustrate the distribution of scores by timepoint. Error bars are 95% confidence intervals, but because the sample is so large they appear to be super-imposed.

The summary results for the tests of difference in mean scores from pre- to post-intervention are presented in Table 2 and illustrated in Figure 2. Following training, participants (*n* = 2,105) reported strong effects from baseline for increased mindfulness (difference: 8.41 [95% CI 8.02, 8.79], *d* = 1.16), and lower perceived stress (difference: 6.18 [95%CI 5.86, 6.50], *d* = 1.00), and a small increase in work engagement (difference: 0.30 [95% CI 0.24, 0.35], *d* = 0.29). The UWES sub-scale scores were consistent in the direction of change, with means for vigor (difference: 0.44 [95% CI 0.38, 0.50]), dedication (difference: 0.25 [95% CI 0.19, 0.32]) and absorption (difference: 0.19 [95% CI 0.13, 0.25]) showing improvement at post-intervention.

**Table 2:** Future Learn MOOC–results for stress, mindfulness, and work engagement following participation in mindfulness training.

**Outcome**	***n***	**Pre-intervention**		***n***	**Post-intervention**		**Difference in means**					**Cohen's d**
		**M**	**SD**		**M**	**SD**	**Difference**	**95% CI**		**t**	***p*-value**	
PSS-10	2,105	18.95	6.55	2,045	12.73	5.58	6.18	5.86	6.50	38.16	<0.001	1.00
FMI	2,087	31.52	7.80	2,067	39.99	6.73	8.41	8.02	8.79	42.83	<0.001	1.16
UWES total	2,081	3.62	1.13	1,958	3.95	1.11	0.30	0.24	0.35	10.41	<0.001	0.29
UWES vigor	2,080	3.56	1.15	1,957	4.03	1.11	0.44	0.38	0.50	14.99	<0.001	0.42
UWES dedication	2,080	3.75	1.34	1,957	4.03	1.32	0.25	0.19	0.32	7.52	<0.001	0.21
UWES absorption	2,081	3.58	1.16	1,957	3.80	1.14	0.19	0.13	0.25	6.38	<0.001	0.19

*PSS, Perceived Stress Scale 10-item (range: 0–40); FMI, Freiburg Mindfulness Inventory (range 14–56); UWES, Utrecht Work Engagement Scale (range 1–6). t-test set with α = 0.05. Cohen's d: within group standardized mean difference effect estimate (Pre to Post-intervention)*.

### *Post-hoc* Analyses

Perceived stress following exposure to the MWPP-MOOC (total PSS_*post*_) was decreased by an expected *d* = −0.48 [95%CI −0.52, −0.44] for each unit increase in mindfulness (FMI_*post*_*-*FMI_*pre*_), *p* < 0.001, adjusted for pre-intervention PSS scores. Similarly, work engagement following exposure to MWPP-MOOC (mean UWES_*post*_) was increased by an expected *d* = 0.45 [95% CI 0.41, 0.50] for each unit increase in mindfulness, *p* < 0.001, adjusted for pre-intervention UWES scores.

To investigate whether the different performance of the UWES dimensions is an artifact of the instrument we inspected the internal consistency data for the whole instrument and at subscale level. Cronbach's test results indicate UWES16 had a poor fit within the absorption subscale, with a considerably lower item:scale correlation (r.cor = 0.44) than the other included items (range r.cor = 0.68–0.86). Whole scale consistency without this item was marginally stronger when it was removed (α = 0.88) than when it was included (α = 0.86). In our longitudinal analyses, all the individual UWES items correlated positively with change in mindfulness, except for the UWES16 item (see Table 3). Further, sensitivity analyses showed the difference between pre and post UWES overall scores returned stronger results without UWES16 (difference = 0.34, t = 11.451, p <0.001) than when this item was included (difference = 0.30, *t* = 10.408, *p* < 0.001). The same was true at subscale level, with a stronger pre-post difference in the absorption mean when UWES16 was excluded (difference = 0.29; *t* = 9.19, *p* < 0.001), than when it was included (difference = 0.19, *t* = 0.64, *p* < 0.001).

**Table 3:** Test of associations between FMI change and UWES items.

	**FMI mean change**
FMI mean change	1
UWES 1 change	0.41
UWES 2 change	0.37
UWES 3 change	0.33
UWES 4 change	0.43
UWES 5 change	0.37
UWES 6 change	0.33
UWES 7 change	0.38
UWES 8 change	0.35
UWES 9 change	0.31
UWES 10 change	0.34
UWES 11 change	0.34
UWES 12 change	0.29
UWES 13 change	0.23
UWES 14 change	0.29
UWES 15 change	0.39
UWES 16 change	0
UWES 17 change	0.26

*Spearmans' r, n = 1,736. UWES, Utrecht Work Engagement Scale; FMI, Freiburg Mindfulness Inventory*.

## Discussion

This paper explored cross-sectional relationships between mindfulness, perceived stress and work engagement in a very large sample of self-selecting adult learners from 130 countries. Cross sectional analyses used data from participants enrolled in, but not yet commenced, the MWPP-MOOC (Aim 1). Longitudinal analyses examined observational pre-post changes in the study variables amongst participants who provided data at both timepoints (Aim 2). Results for Aim 1 support mindfulness as a predictor of both stress and work engagement, and that 73% of the effect of mindfulness on work engagement is direct, with the remaining effect mediated by lower perceived stress. Results for Aim 2 supported the findings from Aim 1, with participants reporting higher mindfulness and work engagement and lower stress following mindfulness training.

### Is Mindfulness a Predictor of Stress and Engagement?

Each unit increase in mindfulness predicted a 0.52 (standardized) unit decrease in stress. This translates to a seven-point decrease in perceived stress on the non-standardized scale and indicates a strong inverse relationship between mindfulness and stress. This finding is in keeping with, and adds weight to, previous epidemiological evidence from smaller samples and clearly shows higher mindfulness correlates with lower stress (Palmer and Rodger, 2009; Bränström et al., 2011; Atanes et al., 2015; Zimmaro et al., 2016).

Mindfulness was also shown to be moderately and positively correlated with work engagement. The overall UWES score is the average of subscale means: vigor, dedication and absorption. Results of regression analyses showed a unit increase in mindfulness predicted a small but statistically significant increase in overall engagement. Previous research has found a similar pattern when testing the relationship between two mindfulness measures and work engagement (Kotzé and Nel, 2016). However, these authors found the mindfulness instrument used in the present study (FMI; Walach et al., 2006) had a weaker relationship with engagement than the Mindful Attention and Awareness Scale (MAAS; Brown and Ryan, 2003), and did not include the absorption dimension of the UWES in analyses. Participant comments in the moderated forums of a positive relationship between mindfulness and work engagement was observed whether “work” referred to paid work, study, caring for others, or engagement with hobbies (data not shown). Participants reported being more engaged with the activities in daily life, whatever they happened to be.

Mindfulness may help to improve work engagement because it fosters the ability to self-regulate automatic defensive or avoidant reactions to the distress and challenges that arise in the workplace (Malinowski and Lim, 2015). It helps people disengage or detach from the unhelpful attentional and cognitive patterns that reinforce distress, thus allowing fuller engagement with work and other valued activities in daily life. This is supported by the results of our path analysis, which showed that stress partially mediates the effect of mindfulness on engagement and that the majority of the mindfulness:engagement relationship was direct. Our results indicate it may be the qualities of mindfulness itself, rather than its effects on stress, that predominantly drive increased work engagement.

In previous research, Leroy et al. (2013) found the effects of mindfulness on engagement were mediated by authentic functioning, which is linked to self-awareness and self-regulatory capacities (Avolio and Gardner, 2005). Further, the construct of psychological capital, which is a determinant of work engagement and incorporates hope, optimism, self-efficacy and resilience, has been shown to have a clear positive relationship with mindfulness (Avey et al., 2008). The qualities of mindfulness (attentional control, awareness and acceptance) may therefore yield additional, independent benefits on positive work-related performance outcomes such as engagement.

Higher work engagement was also predicted by lower perceived stress in our cross-sectional analyses. One explanation may be that people who are stressed struggle to engage fully with work. This is well-established in occupational health psychology theory and research (Hargrove et al., 2011; Bakker and Demerouti, 2017) and is a key driver of the quest for effective workplace stress management interventions (Bhui et al., 2012). Research has found that stress interferes with working memory capacity, which limits performance (Ashcraft and Kirk, 2001). People who are stressed have difficulty focusing and find themselves getting caught in modes of thinking that perpetuate stress, such as worry and rumination (Ganster and Rosen, 2013). When sustained in this way, stress is known to lead to burnout (Iacovides et al., 2003) and other factors negatively associated with work engagement and performance (Cooper and Dewe, 2008). Our results support the potential of mindfulness training to ameliorate perceived stress and yield independent positive effects on work engagement (Vonderlin et al., 2020).

### Post-intervention Changes in Mindfulness, Stress, and Engagement

Changes in mindfulness, perceived stress and work engagement were assessed following the 6-week MWPP-MOOC. Overall, our observational data indicated significant beneficial changes on all three of the studied variables. A strong positive effect was observed in mindfulness following training. This concurs with a wealth of previous research demonstrating that mindfulness training leads to increased trait mindfulness (e.g., Carmody and Baer, 2008; Shapiro et al., 2008) and that online MBIs can be effective (Morledge et al., 2013; Spijkerman et al., 2016; Cavanagh et al., 2018). Our findings also provide important early evidence in support of the massive open online course (MOOC) format for reaching a large number of people and teaching mindfulness and associated practices (Hodge, 2016).

The results observed for perceived stress are consistent with a large body of literature that shows mindfulness training, delivered either in person or online, significantly reduces self-reported levels of stress in various populations (Chiesa and Serretti, 2009; Khoury et al., 2013; Cavanagh et al., 2018; Bartlett et al., 2019). The primary mechanisms involved may be improved attentional control and increased acceptance of whatever is experienced, skills that are explicitly taught in mindfulness training (Creswell and Lindsay, 2014). That is, mindfulness encourages one to pay full attention to moment-by-moment experience, rather than becoming caught in worry or rumination. This reduces amygdala activation, thereby reducing overall levels of stress (Creswell and Lindsay, 2014; Taren et al., 2015). Research shows that increases in mindfulness tend to precede decreases in perceived stress, suggesting that increased trait mindfulness may mediate the relationship between mindfulness training and stress (Baer et al., 2012). This sequential development of outcomes has been identified previously (Garland et al., 2017b) and may explain why stronger associations between stress and mindfulness were found in our cross-sectional analyses, than observed in our longitudinal analyses, where participants' mindfulness skills were newly emergent at the end of training.

Our finding that MWPP-MOOC participants reported higher work engagement after training adds weight to previous research (Leroy et al., 2013; Atkins et al., 2015). Statistically significant improvements were observed on all three dimensions of work engagement, with change in vigor clearly the most pronounced effect. The dominance of vigor may be explained by its correspondence with resilience and vitality, two constructs established as positive correlates of mindfulness (Allen and Kiburz, 2012; Smith, 2014). Dedication also improved, but not to the same extent as vigor. Dedication is potentially and conceptually associated with mindfulness via attentional control and prosocial acting, however these nomological relationships are not well-studied. Absorption was the dimension of engagement least responsive to mindfulness training. It is feasible the UWES absorption construct, as it is currently assessed, represents an inability to let go of work and focus on other important areas of life.

In an attempt to explain the engagement results, we conducted *post-hoc* tests that showed one of the items in the absorption subscale (UWES16: “It is difficult to detach myself from my job”) had a poor fit with the other items in the measure. Further, we found stronger pre-post effects for the overall work engagement and absorption subscale without this item. Our findings support the need for careful consideration of including UWES16 in a positively-oriented measure intended to detect healthy work engagement, and associations with positive, adaptive qualities such as mindfulness. For example, mindfulness training cultivates skills that support the regulation of attention, and is evidenced for *increasing the ability to detach* from absorbing thoughts and to engage more fully with other aspects of one's life (Malinowski, 2013; Li et al., 2018). There may be some construct confusion when work engagement is conceptualized as being interested, motivated and on-task while working, but also as an inability to let it go even when one should. Future research should seek to elucidate the difference between absorption, concentration, acceptance and letting go, and how these may likewise have differential effects of work engagement.

Work engagement is an important indicator of employee wellbeing and organizational performance (Seppälä et al., 2009; Xanthopoulou et al., 2009) but this construct has been infrequently studied in mindfulness intervention research (Malinowski and Lim, 2015; Bartlett et al., 2019). Recent evidence on the face-to-face delivery of MBIs such as MBSR indicate that it can be effective in increasing resilience (Klatt et al., 2021) and work engagement at the same time as reducing burnout (Lo et al., 2021) but it is currently unclear whether similar benefits can be derived from scalable and affordable brief online MBIs. Our findings that mindfulness training is clearly associated with higher engagement—and in particular, patterns of adaptive engagement—thus offer an additional contribution to the literature but further research and different methodology are required to establish whether this relationship is causal. As previously mentioned, work engagement has three dimensions of vigor, dedication and absorption and it commonly has an inverse relationship with burnout. Many of the work-related health benefits of mindfulness are likely because of its ability to reduce burnout as well as other negative outcomes of demanding or insecure work environments like action crises (Marion-Jetten et al., 2021). This interrelationship between burnout and work engagement is important with one review concluding that burnout is more strongly related to health outcomes, whereas work engagement is more strongly related to motivational outcomes (Bakker et al., 2014).

Based on emerging evidence, it behooves companies to introduce training in generic but adaptable skills like mindfulness to reduce burnout, support dealing with work demands and action crises at the same time as enhancing innovative potential and work engagement. The onus of this responsibility must, however, not fall on the shoulders of individual employees alone who may have found themselves working in dysfunctional and unhealthy work environments. It must be a collective responsibility shared by individual workers and employers to cooperatively create healthy workplace practices and culture.

This paper reports procedures and results of two research questions investigating the relationship between mindfulness, stress and work engagement. Our large, heterogeneous sample of adult learners provided sufficient data for us to conclude that high levels of trait mindfulness is likely to predict substantially lower perceived stress and small, beneficial increments in work engagement. Of the three dimensions of work engagement assessed, mindfulness yields the strongest influence on vigor. While stress partially mediates the relationship between mindfulness and work engagement, most of the effect is directly attributable to mindfulness. Further, we found the MWPP-MOOC format to be effective for teaching and learning mindfulness, and that participants reported higher work engagement and lower stress immediately after the program.

### Limitations and Future Directions

MOOCs generally tend to have high attrition rates. The high attrition in survey responses between baseline and post-intervention means it is possible that positively biased people provided full data, and that the effect estimates may have been inflated as a result. Further, there was no unique identifying variable to support linking of demographic, pre- and post-intervention data, and to enable participant characteristics to be linked with outcome data for profile analyses. The absence of a unique identifier meant demographic data was not linked with outcome data and several thousand cases could not be confidently matched across time points. The potential for longitudinal analyses on the full data set was not realized.

The degree of covariance between items across all scales was less than the 50% threshold recommended by Harman's test (Fuller et al., 2016). This suggests there was not severe common method variance, although this bias must be acknowledged. However, all findings were theoretically supported, and the use of validated scales provide ex-ante control for common method bias (Orben and Lakens, 2020).

The use of a control group in our longitudinal study, and access to linked demographic and course engagement data would have enabled more rigorous and in-depth analyses and provided greater confidence that observed changes following the MWPP-MOOC could be attributed to participation. For example, adherence to the course's didactic, interaction and practice elements would enable examination of dose-response relationships and identify process variables that may be responsible for differential effects experienced by participants. We did not measure adherence, or the amount and type of mindfulness practice undertaken. This decision was made to minimize participant burden, however future research should include these valuable metrics as adherence has been shown to be an important predictor of outcomes from mindfulness programs (Carmody and Baer, 2008, 2009; Bowen and Kurz, 2012) and that there may be a clear dose/response relationships (e.g., Strohmaier, 2020). Finally, follow-up data would be useful to explore the longevity of training effects and help determine a “sensitive window” for effects to diminish, or further develop. Such information would help guide the way future MBIs are delivered online to ensure maximum impact.

## Data Availability Statement

The datasets presented in this article are not readily available because at the time of collecting the data ethics approval to make it openly available was not sought or given. Requests to access the datasets should be directed to Craig Hassed, craig.hassed@monash.edu.

## Ethics Statement

This study was undertaken in accordance with ethical standards of the Monash University Human Research Ethics Committee (REF: 18105). The manuscript presents no animal studies or identifiable human images. Informed consent was obtained from all participants in the study. Written informed consent for participation was not required for this study in accordance with the national legislation and the institutional requirements.

## Author Contributions

LB conducted the analyses and drafted the manuscript. M-JB and AB designed the analytic approach, contributed to running the analyses, and interpreting the results, including figures. CH and RC conceived and designed the study and provided the course description and data for analyses. All authors contributed to the final version of the manuscript.

## Conflict of Interest

CH and RC are the developers and lead facilitators of the course being evaluated. Neither author receives financial remuneration from either of those roles. The remaining authors declare that the research was conducted in the absence of any commercial or financial relationships that could be construed as a potential conflict of interest.

## Publisher's Note

All claims expressed in this article are solely those of the authors and do not necessarily represent those of their affiliated organizations, or those of the publisher, the editors and the reviewers. Any product that may be evaluated in this article, or claim that may be made by its manufacturer, is not guaranteed or endorsed by the publisher.
