# Supplementary material for: Mindfulness Is Associated With Lower Stress and Higher Work Engagement in a Large Sample of MOOC Participants
Source: Front Psychol. 2021 Sep 10;12:724126. doi: 10.3389/fpsyg.2021.724126 (PMC8461060; doi:10.3389/fpsyg.2021.724126)
Supplement: Supplementary file 1 [file Data_Sheet_1.docx]

Supplementary Material

Correspondence to [Craig.Hassed@monash.edu](mailto:Craig.Hassed@monash.edu)

**Mindfulness for Wellbeing and Peak Performance Massive Open Online Course (available via FutureLearn).**

The developers of the Mindfulness for Wellbeing and Peak Performance MOOC (MWPP-MOOC) have medical (C. Hassed) and psychology (R. Chambers) backgrounds and each has decades of experience in developing, contextualising and delivering mindfulness training in educational, workplace and community settings. Both have ongoing positions at a large Australian university where they deliver mindfulness training programs to over 6,500 people per year as well as working with groups outside the university.

The MWPP-MOOC is iterative, interactive and experiential and develops a practical understanding of mindfulness and how to apply it personally, professionally and/or academically. It is based on mindfulness principles consistent with MBSR or MBCT but it is not called by either of those names because of the shorter length of the course, briefer duration of recommended daily meditation practice, absence of mindful yoga and the method of online delivery. Key elements of the program each week include short videos outlining key principles and how to apply them, guided meditation practices, key readings, links to further resources, an evidence-base, weekly quizzes, discussion boards monitored and supported by trained psychologists, and weekly feedback videos.

We don’t call the program ‘treatment’ or aim at therapeutic outcomes although some participants do mention in the discussion forums that they have a history of conditions like anxiety or depression. We recommend that participants do not reveal any very personal information on these forums. Although participants do often comment that they find the MOOC of therapeutic value, we strongly recommend that participants move through the program gently and with care, and that if they ever experience any significant mental health issues or symptoms that they cease or defer the MOOC and seek more personalised guidance and support from a trained mental health professional.

**MWPP Course Structure**

Week 1: Introducing mindfulness

1. Getting started
   - This week, get started with mindfulness. Learn more about what it means to be mindful and unmindful, why mindfulness matters to your personal and professional life, and what you’re going to do in this course.
2. Mindfulness meditation
   - Start the course with a very brief mindfulness meditation, the Comma.
3. The problem of 'unmindfulness'
   - Learn more about the costs associated with being unmindful.
4. Feedback from the educators
   - Watch the course educators provide feedback on various issues and questions that have arisen from discussions throughout the week.

Week 2: Mindfulness and mindful stress reduction

1. Introduction
   - Learn about the stress response and its implications for wellbeing and performance.
2. Cultivating gentleness
   - Explore a central quality of mindfulness - gentleness.
3. The power of curiosity
   - Rediscover the innate human quality of curiosity and notice the benefits this has for your wellbeing and cognitive performance.
4. Mindfulness in daily life
   - Discover where you can bring mindfulness into your daily activities.
5. Feedback from the educators
   - Watch the course educators provide feedback on various issues and questions that have arisen from discussions throughout the week.

Week 3: Improving work and study performance

1. Introduction
   - Learn more about mindfulness and why attention matters so much for performance and learning.
2. The cognitive practices of mindfulness
   - Explore how mindfulness meditation can help us to cultivate the cognitive skills to manage stress, learn and perform better.
3. The power of unitasking
   - Learn more about multitasking, unitasking and efficient attention switching, and more.
4. Mindfulness for productivity
   - Learn how you can apply mindfulness to be more productive in your life.
5. Feedback from the educators
   - Watch the course educators provide feedback on various issues and questions that have arisen from discussions throughout the week.

Week 4: Mindfulness as a way of life

1. Introduction
   - Learn about the benefits of self-compassion for wellbeing and performance.
2. Mindfulness and emotions
   - Learn how mindfulness can help you to better understand and manage your emotions.
3. Mindful eating
   - Learn how you can apply mindfulness to the way you engage with the food you eat.
4. Mindfulness for creativity
   - Learn how you can apply mindfulness to boost your creativity, and then practise a meditation.
5. Explore, develop and maintain mindfulness practices
   - Learn more about how you can explore, develop and maintain mindfulness practices in your life by exploring resources and other learning opportunities that are available outside the course.
6. Feedback from the educators
   - Watch the course educators provide feedback on various issues and questions that have arisen from discussions throughout the week.

Week 5: Communication and relationships

1. Welcome to the week
   - This week, get started with expanding and maintaining mindfulness in your life. Learn how you can apply mindfulness principles to improve your communication and practise a mindful listening meditation exercise.
2. Expanding and maintaining your practice
   - Get some advice on mindfulness, and learn more about expanding and maintaining your mindfulness practice.
3. Mindfulness meditation
   - Practise the listening meditation.
4. Mindful communication
   - Learn more about how you can be more mindful in the way you communicate.
5. Mindful relationships
   - This week, learn how mindfulness can improve your relationship with yourself, and others.
6. Empathy and compassion
   - Explore how you can be more compassionate with others and participate in an meditative learning exercise helping to differentiate between empathy and compassion.
   - Practise these meditations to better cultivate kindness and compassion.
7. Weekly feedback from the educators
   - Watch the teachers provide feedback on various topics and questions that have arisen from discussions throughout the week.

Week 6: Cultivating self-compassion and emotional health

1. Welcome to the week
   - This week, learn how you can bring mindfulness to self-compassion and performance, and to pain.
2. Self-compassion and performance
   - Learn how self-compassion can impact your performance, and practise a meditation.
3. The big picture
   - This week, learn about the 'big picture' and how you can embed mindfulness into all aspects of your life.
4. Mindfulness meditation
   - Practise the choiceless awareness meditation.
5. Ethics, values and goals
   - Find out how mindfulness can help you connect with your ethics, values and goals to help you lead a better life.
6. Everyday mindfulness
   - Learn how you can apply mindfulness to the way you eat, respond to news in the media, and more.
7. End of course
   - Watch the teachers provide their final thoughts and feedback on various topics and questions that have arisen from discussions throughout the week, and access resources that you can use to help you to maintain a mindful life.
